# Trust Execution Environment and Multi-party Computation for Blockchain e-Health Systems

**DOI:** 10.1007/978-3-030-51517-1_24

**Published:** 2020-05-31

**Authors:** Feriel Yahmed, Mohamed Abid

**Affiliations:** 8 Digital Research Centre of Sfax, Sfax, Tunisia; 9 Institut Mines-Télécom, CNRS, Paris, France; 10 Université de Sherbrooke, Sherbrooke, QC Canada; 11 Digital Research Centre of Sfax, Sfax, Tunisia; 12 University of Sfax, Sfax, Tunisia; Unit Hatem Bettaher IRESCOMATH, University of Gabes, Gabes, Tunisia

**Keywords:** E-health, MPC (Multi-party computation), BC (Blockchain), TEE (Trust Execution Environment) and Smart Contract (SC), IPFS (Interplanetary File System)

## Abstract

Blockchain is a rich and attractive domain for researchers since it is independent of “third party” such as Bank or government. This “open” phenomenon does not respect all the security criteria such as private data protection and confidentiality; hence, we cannot trust this approach despite its contributions. Blockchain technology has gained considerable progress in recent years in fields such as e-health. The medical data contains personal and sensitive information that must be preserved. The current Blockchain systems suffer from serious practical limitations, e.g. poor performance, high-energy consumption and lack of confidentiality. On the other hand, *Trust Execution Environment* TEE is imperfect; it is based on the centralization of data. To avoid data centralization and its limitations, an approach based on collecting the necessary data from distributed database is presented in this paper. Our goals are to protect the user’s privacy and to execute it in TEE combined with *Multi*-*party computation* MPC. We proof by security analysis that our new solution meets the fundamental criteria of security such as confidentiality and privacy.

## Introduction

Technological evolution is bringing a profound change to the core of business. Nowadays IT(Information Technology) is not only a productivity tool but also a means of administration and management. It is becoming a strategic and a necessary mean to manage the evolutionary processes of the company’s business lines. Therefore, the field of information and communication technologies has become one of the pillars of business.

The information system is an essential element for the company; hence, its innovation must be almost permanent and exploits to the best the new technologies. New network technologies open up new potential for communication and data exchange in different geographical areas. This context has motivated the IT community to take an interest in distributed architectures such as the Blockchain.

Modern systems like Blockchain have become increasingly complex, open, connected and are leading to new challenges. User requirements for security are increasingly demanding. The Blockchain has affected several sectors such as finance, health care, public services, electronic voting, music and the government sector. The reason for this enhanced interest is the disappearance of the trusted medium, to operate in a decentralized manner with an acceptable degree of confidence.

The IT community hails the Blockchain as the next great technological innovation. According to Marc Andreessen, co-founder of Netscape and co-writer of Mosaic the Blockchain: “When we sit here in 20 years, we will talk about [Bitcoin and Blockchain technology] the way we talk about the internet today” [1].

### Blockchain

**Fig. 1.**
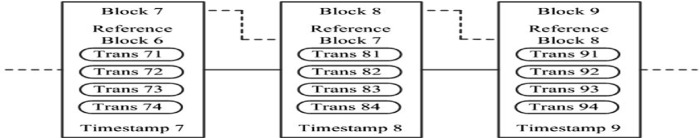
Blockchain structure [17]

The Blockchain is a new technology for storing and transmitting data in a secure and transparent way, it works without a central control body. This technology takes the form of a transaction log in a peer-to-peer P2P network. These transactions grouped together in the form of blocks, which are linked together. Each block contains data, the hash of the previous block, and a time stamp. Figure 1 represents an example of Blockchain structure.

All network nodes back up and verify the data stored in the Blockchain, and consequently provides a strong resilience against attacks that can tamper the integrity of the data.

As this great feature leads to a Blockchain-based implementation of smart contract platforms such as Ethereum [2], several developers have been attracted to build decentralized applications using smart contracts that avoid the need for a central server to manage and maintain the data [3].

**Fig. 2.**
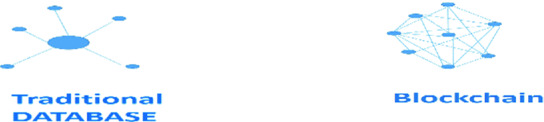
Traditional DATABASE VS Blockchain

The first cryptocurrency based on a Blockchain was Bitcoin in 2008 [7], however the Blockchain has evolved to meet and serve a variety of purposes. The difference between a traditional database and the Blockchain is essentially the storage policy. The Blockchain resides on computer networks However, databases are stored on centralized servers (see Fig. 2). Each one of them has its own advantages and limitations.

### Smart Contract

Szabo first introduced the term *smart contract* in 1994, where the smart contract is defined as “a computerized transaction protocol that executes the terms of a contract” [4].

Smart contracts are compiled as byte codes and executed in EVM (Ethereum Virtual Machine) located in miners’ computers, which is very similar to Java executed in JVM. When the smart contract operates, it must be packaged by the miner and written into the Blockchain. Each Blockchain in Ethereum has various functions and purposes [5]. Compared with traditional contract, a smart contract is an executable code stored and running in Blockchain. The smart contract may execute independently and automatically without third parties, and these running results are irreversible on Blockchain and are traceable by each participant. The main features of smart contract are given as follows [6]: stability and deterministic features, the same input always produces the same output. Because smart contracts are executable codes stored in Blockchain, every network participant can inspect them. Meanwhile, all the interactions with a smart contract occur via signed messages on the Blockchain and thus every participant can verify and trace the contract’s operations.

The structure of this paper is as follows. Section 2 presents a state of the art by analyzing the current situation and motivation. Section 3 describes the steps of the new solution and an e-health use-case. We present the security analysis of the proposed solution in Sect. 4. Conclusion is drawn in Sect. 5.

## State of the Art

We have conducted an intensive research to get the state of the art of Blockchain and smart contracts applications. In the following, we present the existing solutions based on the technologies chosen by researchers.

### Centralized Database

The researchers in [8] propose a solution for digitizing certificates, in university use case, in order to improve the conditions and make life much easier using the Blockchain and intelligent contracts. Therefore, it will be possible to have a certificate, wherever the student is and whatever the time, with full security since the access to the data will be done only when people are authorized.

It is true that this solution has contributions in terms of time and speed. However, in our opinion, it does not ensure total security since it puts in danger the private data when they are published in the Blockchain. In addition, the weak point of the solution is the centralization since the data are recorded in the database of the university and if it is broken down, nothing can be done.

### PKI Public Key Infrastructure

Existing certificate mechanisms do not dynamically ensure the trustworthiness of a certificate, to solve this weakness Ahmed et al. [9] offer the “smart contract assisted PKI”. This solution manages trust dynamically in a distributed way and provides better trust experience for users. Despite its contribution, this solution neglects the protection of private data.

### TEE Trust Execution Environment

TEE (Trust Execution Environment) [16] is a tamper-proof trusted execution processing environment. It runs on a separate kernel and it can be safely updated.

TEE resists all software and physical attacks. It represents a space for storage execution and secure execution.

In this context, Hawk [10], which is the first TEE, is a smart contract system that provides confidentiality by executing contracts off-chain and posting only zero-knowledge proofs on-chain. The zero-knowledge proofs in Hawk incurs very high computational overhead. Additionally, it was designed for a single compute node called the “manager” which must be trusted for privacy.

There are also some technical inefficiencies such as limited block size and transfer cost. In addition, once the data is stored in the Blockchain, it cannot be modified. This poses certain problems such as falsification during the execution of the contract. In some cases, the contract needs data in real time. The most relevant solution is to store the data off-chain and choose another execution platform more secure and more relevant.

To ensure confidentiality and private data protection, Rifi et al. [11] offer a solution that combines two technologies: *Trust Execution Environment* TEE and Blockchain, from where data is stored and executed in TEE. It is a platform that ensures data integrity; confidentiality and protection, but it is based on the centralization of data storage. In [11], authors concluded that in order to apply Blockchain technology to E-Health, it should be public, and has three main keys: scalability, secure access to medical data, and data privacy. In this article, the researchers prove that the Blockchain does not have good performances in terms of storage. Therefore, they proposed to store the data off-chain using the IPFS (*Interplanetary File System)*. The new Blockchain technology [15] applied in e-Health identifies new ways to share the distributed view of health data and promotes the advancement of precision medicine, improving health and preventing diseases.

### IPFS

IPFS [12] (*Interplanetary File system)* is a decentralized file sharing platform. It identifies files by their contents. To retrieve file locations and connectivity information from the nodes, this system uses Distributed Hash Table (DHT).

As described in [13], the DHT is primarily a distributed key value store. It uses node identifiers and keys. They must have the same length and a distance metric to easily store and retrieve the information. A node tries to find the nodes in its vicinity, when searching a value and a key. To do this; it uses buckets to keep track of the nodes in the network. The spaces are organized so that each node in the network has precise information about its immediate environment.

Blockchain and IPFS [14] are based on similar concepts of decentralized networks. However, each one of them has its own characteristics. IPFS is a file-sharing system that chops its files. The search for files within IPFS is based on these hashes. Blockchain and IPFS perform very different tasks for their users. It is possible to store files in IPFS while the hashes are stored in Blockchain.

As discussed before, the TTE has many advantages but it is not based on multi-party computation. Therefore, to eliminate the centralization of data, we try to link between IPFS, TEE and the Blockchain to have efficient results that respect the security rules.

**Fig. 3.**
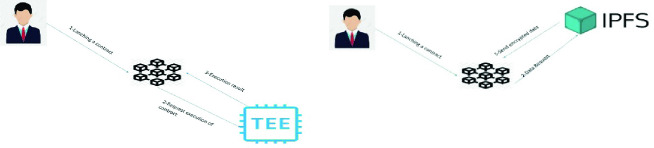
Smart contract with TEE VS Smart contract with IPFS

The use of TEE allows the user to store his/her data in TEE and to execute his/her smart contract. To access to the latter, the user must enter his/her public hash key; TEE compares it with the list of public hash keys; if it is compatible with a public hash key, he/she can access to it. The Blockchain is used to transfer smart contract from user to TEE (see Fig. 3 Smart contract with TEE). To import data from IPFS, the user should put the IPFS Hashes and a time stamp in the smart contract (see Fig. 3 Smart contract with IPFS). As a conclusion, executing smart contract in Blockchain suffers from many problems such as poor performance, high-energy consumption.

## The Proposed Solution

With the technical progress, many technologies are developing, a huge amount of exchanged data will appear, and the exchange of data is carried out from different locations and different sources. In order to ensure all these criteria, we must have a solution that provides: Confidentiality, Authenticity, Integrity, Decentralization and privacy in two phases: Data Storage and Smart contract’ execution.

### Architecture and Security Parameters

**Fig. 4.**
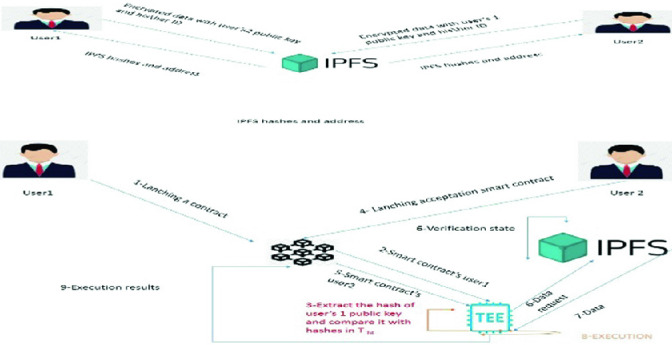
Proposed architecture

Figure 4 presents the architecture of the new solution which is composed by two phases:

- Data Storage

In this phase, the user stores his\her encrypted data in different places. To access to it, we need to process some cryptographic steps.

b) Smart contract's execution

Before this operation, we need to transfer the smart contract using Blockchain. After that, the TEE “imposes” some protocols to access to it. Also, TEE controls missing data and imports them from DDB (Distributed Data Base) The approach proposed in Article [18] can be used to collect data either from IPFS or from users. After that, it executes the smart contract and returns the result to the Blockchain.

Table 1 contains all the security parameters of the communicating parties in e-health scenario.

**Table 1.** Security parameters

Parameters	Description
H_IPFS_	Hashes IPFS
H_Kpub_	Hashes public key
K_pub_	Public key
K_priv_	Private key
@_IPFS_	IPFS address
T_ht_	Hashes table in TEE
L_DD_	List of demanded data
EN_Kpub_	Encrypt with public key

### Steps of the New Solution

Next, we describe, step by step, the exchanges in the new solution:

- *User’s subscription (Data Storage)*

A user accesses an IPFS and stores his/her encrypted message with homomorphic cryptography. The encryption ensures data security since only authorized persons have the right to access encrypted data. IPFS sends to the user its @_IPFS_ and H_IPFS_.

1. User 1 sends his\her Id and EN_Kpubuser2_(Data) to the IPFS.
2. IPFS sends @_IPFS_ and H_IPFS_ to User2
3. User 2 sends his\her Id and EN_Kpubuser1_(Data) to the IPFS.
4. IPFS sends @_IPFS_ and H_IPFS_ to User1
5. *Smart contract execution*
  1. User 1 signs his\her smart contract with his/her private key K_priv_ (this smart contract demands @_IPFS_ and H_IPFS_ from the acceptation smart contract) and sends it to the Blockchain.
  2. The Blockchain sends the signed Smart Contract to the TEE
  3. TEE extracts the hash H_kpub_ and compares it with the hashes in T_ht_
  4. User 2 signs his\her contract that contains his\her @_IPFS_ and H_IPFS_ with his\her K _priv_ and sends it to the Blockchain.
  5. The Blockchain sends the signed Smart contract to the TEE. The latter extracts the H_Kpub_ of User 2 and compares it with the hashes in T_h._ After that, it builds a file that contain missing data.
  6. TEE sends the file that contains the L_DD_ (List of demanded data), @_IPFS_, H_IPFS_ and timestamp (from acceptation smart contract) to IPFS
  7. IPFS verifies the TEE request that contains @_IPFS_, H_IPFS_, L _DD_ and timestamp after that it sends missing data to the TEE.
  8. TEE executes the smart contract
  9. TEE sends the executed results to the Blockchain.

### E-Health Use Case

**Fig. 5.**
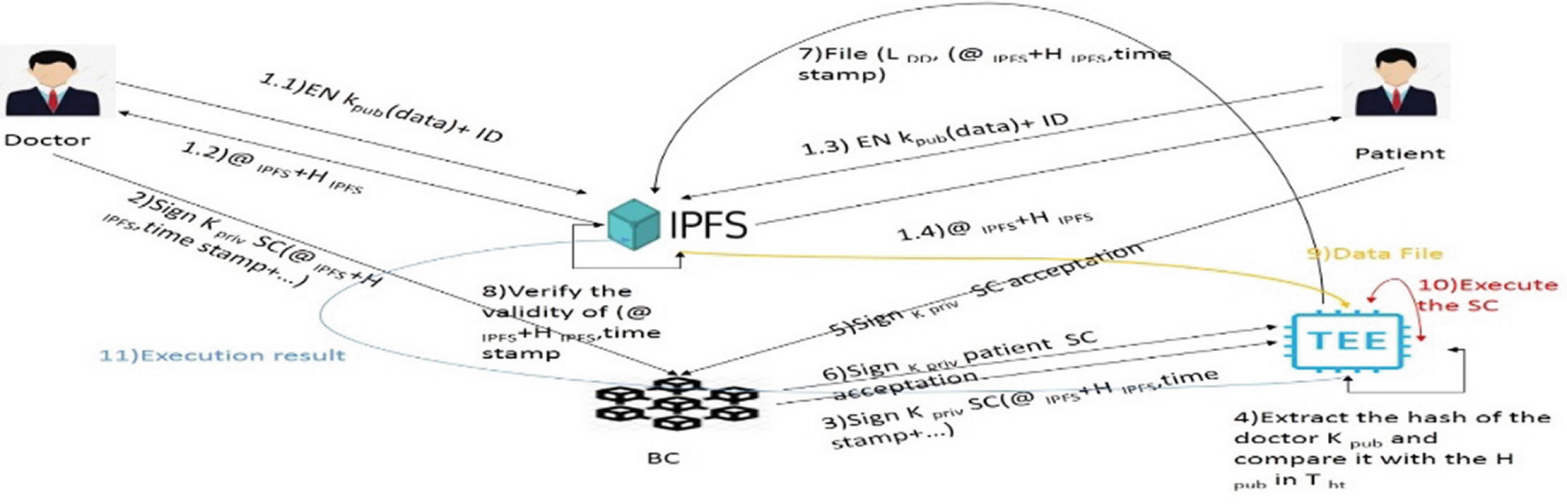
All the exchanges in e-health scenario

We present in the following the steps of our solution when applied to e-health scenario. In this use case, we have two actors, the doctor and the patient. Figure 5 shows the steps of this use case.

1.1, 1.2, 1.3 and 1.4: Both doctor and patient send EN_Kpub_ (data) + ID and receive @_IPFS_ + H_IPFS_

- 2 - The doctor sends his/her ID and signed smart contract to the Blockchain.
- 3 - The Blockchain sends this smart contract to TEE
- 4 - The TEE receives the smart contract, after it verifies the state of the doctor. Then, it extracts the public key of the doctor and calculates its hash and compares it with the hashes in T_ht_
- 5 - The patient signs smart contract acceptation with his\her private key and sends it to the TEE using Blockchain.
- 6 - The Blockchain sends the signed SC patient with K_priv_ to the TEE.
- 7 - TEE creates a file that contains a list of demanded data with IPFS hashes, _@IPFS_ and the time stamp and send it to the IPFS.
- 8 - IPFS verifies the validity of (@_IPFS_ + H_IPFS_, time stamp).
- 9 and 10 - TEE executes the SC and returns the execution result to the Blockchain.

## Security Analysis

Table 2 presents a comparison between three previous solutions and our proposed solution. We observe that the others solution does not respect all the criteria of security.

**Table 2.** Comparison approaches

Articles	Confidentiality	Privacy	Authenticity	Integrity	Decentralization
Hawk [10]	+	+	+	+	−
BC and SC for digital certificate [8]	+	−	+	+	+
Towards using BC technology for e-Health data access management [11]	+	−	+	+	+
Our solution	+	+	+	+	+

When using this approach, the 5 key criteria of security are guaranteed or maintained. in short, user data can only be modified, accessed or deleted by authorized persons (integrity; privacy and integrity).

The IPFS contains the data in a homomorphic way (decentralization) and the execution in TEE will be based on the encrypted data, without forgetting that the TEE and IPFS platforms only allow access to authorized persons.

## Conclusion

The Blockchain technology has reached a great boom in many sectors such as e-health, e-commerce; e-vote… The main important Blockchain [11] characteristics are: Transparency, no need for third parties and instant access to data since it is replicated on all nodes. To create a secure smart contract based on Blockchain, a new solution is presented which combines three technologies: Blockchain, TEE, and IPFS in order to take advantage of their benefits. This approach is based on collecting the necessary data from distributed database while protecting the user’s privacy and executing it in TEE. This approach meets the various security criteria such as confidentiality, authentication, integrity, decentralization and protection of private data. this approach can be applied in different domains such as e-health to show the strong points of this one in the e-health domain it is necessary to link it with the Internet of Things domain [19].

As future work, we need to implement the new solution in order to study its feasibility and measure its performance.
